# Enhanced Protein Damage Clearance Induces Broad Drug Resistance in Multitype of Cancers Revealed by an Evolution Drug‐Resistant Model and Genome‐Wide siRNA Screening

**DOI:** 10.1002/advs.202001914

**Published:** 2020-10-11

**Authors:** Fangyuan Shao, Xueying Lyu, Kai Miao, Lisi Xie, Haitao Wang, Hao Xiao, Jie Li, Qiang Chen, Renbo Ding, Ping Chen, Fuqiang Xing, Xu Zhang, Guang‐Hui Luo, Wenli Zhu, Gregory Cheng, Ng Wai Lon, Scott E. Martin, Guanyu Wang, Guokai Chen, Yunlu Dai, Chu‐Xia Deng

**Affiliations:** ^1^ Cancer Center Faculty of Health Sciences University of Macau Macau 999078 China; ^2^ Center for Precision Medicine Research and Training Faculty of Health Sciences University of Macau Macau 999078 China; ^3^ Guangdong Key Laboratory of Animal Breeding and Nutrition Institute of Animal Science Guangdong Academy of Agricultural Sciences Guangzhou 510640 China; ^4^ Department of Biology Southern University of Science and Technology Shenzhen 518055 China; ^5^ Kiang Wu Hospital Macau 820002 China; ^6^ Centro Hospitalar Conde de S. Januário Macau 820004 China; ^7^ Division of Pre‐Clinical Innovation National Center for Advancing Translational Sciences (NCATS) National Institutes of Health Bethesda MD 20892 USA

**Keywords:** broad drug resistance, caspase 3 activation, patient‐derived organoid, proteolysis, RNAi screening

## Abstract

Resistance to therapeutic drugs occurs in virtually all types of cancers, and the tolerance to one drug frequently becomes broad therapy resistance; however, the underlying mechanism remains elusive. Combining a whole whole‐genome‐wide RNA interference screening and an evolutionary drug pressure model with MDA‐MB‐231 cells, it is found that enhanced protein damage clearance and reduced mitochondrial respiratory activity are responsible for cisplatin resistance. Screening drug‐resistant cancer cells and human patient‐derived organoids for breast and colon cancers with many anticancer drugs indicates that activation of mitochondrion protein import surveillance system enhances proteasome activity and minimizes caspase activation, leading to broad drug resistance that can be overcome by co‐treatment with a proteasome inhibitor, bortezomib. It is further demonstrated that cisplatin and bortezomib encapsulated into nanoparticle further enhance their therapeutic efficacy and alleviate side effects induced by drug combination treatment. These data demonstrate a feasibility for eliminating broad drug resistance by targeting its common mechanism to achieve effective therapy for multiple cancers.

## Graphical abstract

Many anticancer drugs can induce protein damage, which is a common way to kill cancer cells. Meanwhile, the damaged proteins trigger mitochondrial protein import surveillance checkpoint which impairs mitochondrial respiration activity, leading to the activation of proteasome and clearance of damaged proteins. This feedback loop triggers broad drug resistance, which is overcome by proteasome inhibitors.

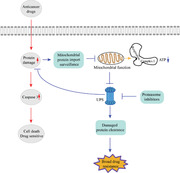

## 1 Introduction

Drug resistance, either due to the lack of original response or gradual loss of responsiveness during chemotherapy, occurs in virtually all kinds of cancers.^[^ ^1^ ^]^ Cancer cells can also become multidrug resistance when they are treated simultaneously or sequentially with several drugs.^[^ ^2^ ^]^ Frequently, cancer cells that develop resistance to one drug also become resistant to some other drugs that are structurally unrelated and to which the patient was not previously exposed.^[^ ^3^ ^]^ In all these cases, cancers become very difficult for therapeutic treatment, which is one of the major reasons for the high mortality in cancer patients.^[^ ^4^ ^]^

Cisplatin is a widely used drug for cancer therapy. It has been increasingly applied for the treatment of breast cancer, especially in the patients who are carriers of BRCA germline mutations.^[^ ^5^ ^]^ However, cisplatin resistance occurs highest among all anticancer drugs, generating a major obstacle for the wide application of cisplatin in cancer therapy.^[^ ^6^ ^]^ It was shown that when non‐small cell lung cancers acquire resistance to cisplatin, they are also refractory to vinorelbine,^[^ ^7^ ^]^ and cisplatin‐resistant ovarian cancer also develops resistance to paclitaxel.^[^ ^8^ ^]^ Numerous studies have revealed that cisplatin resistance may develop through multiple mechanisms, as it binds to many targets both in the nucleus and cytoplasm, although nuclear DNA is believed to be its major target.^[^ ^9^ ^]^ We have also found that cisplatin resistance could be caused by enrichment of cells with higher levels of efflux transporters (ATP7A), which prevents cisplatin from entering the nucleus^[^ ^10^ ^]^ or by activating DNA replication checkpoint that reduces cell proliferation and enables more time for DNA damage repair.^[^ ^11^ ^]^ These findings provided molecular basis for multidrug resistance, although only 56 genes^[^ ^10^ ^]^ and 706 genes^[^ ^11^ ^]^ were screened in these studies, respectively. We hypothesized that upon drug treatment, especially persistent drug stress, cancer cells may experience an evolution process to gradually develop drug resistance for better survival; and such a process may be initially random but gradually enrich for changes that render cells stronger and stronger ability to tolerate drug scrutiny. Identification of these changes would greatly facilitate the development of potent therapies for overcoming drug resistance.

In this study, we have designed two strategies to identify the changes that may endow cells with the strongest drug resistance. We first employed a genome‐wide screening with 64 755 siRNAs targeting 21 585 genes in order to identify all possible changes for modulating cisplatin resistance in the MDA‐MB‐231 human breast cancer cell line, which is already quite resistant to cisplatin.^[^ ^12^ ^]^ We also treated MDA‐MB‐231 cells with increasing concentrations of cisplatin for a prolonged period to enrich cells with acquired resistance through an evolution process. We found that cisplatin resistant cells developed broad drug resistance for as many as 40 of 69 anticancer drugs tested due to enhanced protein damage clearance. We further demonstrate that this common mechanism can be overcome by a specific drug combination that is further enhanced by using a nanoparticle‐mediated delivery system.

## 2 Results

### 2.1 Genome‐Wide RNAi Screening Identifies the Proteasome as a Target for Enhancing Cisplatin Efficacy

**Figure 1:**
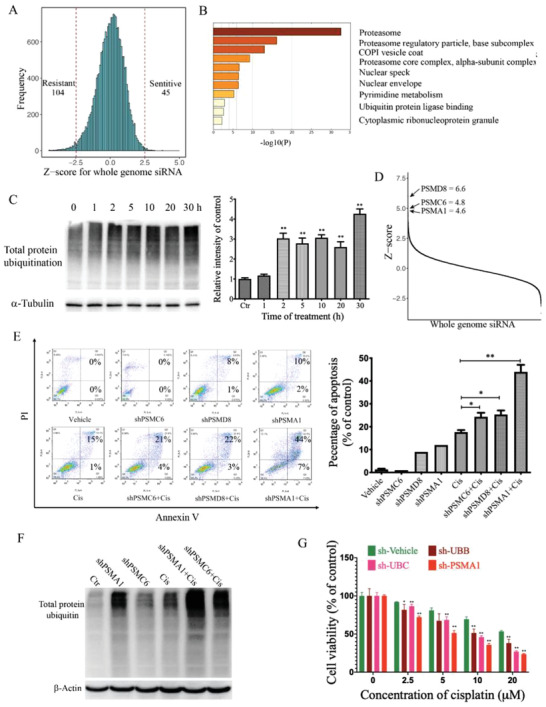
Genome‐wide RNAi screening identifies proteasome as a target for enhancing cisplatin efficacy. A) After RNAi screening, *Z*‐score for each individual gene is calculated as: *z* = (*x* − *μ*)/*σ*, where *x* is the experimental value; *μ* is the median screen value; and *σ* is the standard deviation for the screen, and candidate genes with *Z* score ≧ 2.5 were sorted out as sensitive list, whereas genes with *Z*‐score ≦ −2.5 were defined as resistant genes. B) 45 candidate genes were sorted out in the sensitive list, which increase cisplatin effects. Enrichment analysis of the 45 candidate genes shows proteasome components ranked the first. C) Effects of cisplatin on the total protein ubiquitin level. MDA‐MB‐231 cells are treated with cisplatin as indicated time, and protein total ubiquitin is detected by western blot and gray value was measured for each group of three independent assays. D) The whole genome siRNAs were ranked by *Z*‐score and three top candidates, PSMD8, PSMC6, and PSMA1, are laid out. E) MDA‐MB‐231 cells were transfected with PSMC6, PSMD8, and PSMA1 shRNAs, respectively, then cisplatin treatment for 48 h, cell apoptosis is detected by Annexin‐V‐FITC/PI staining. F) MDA‐MB‐231 cells were treated with cisplatin alone or combined with PSMC6 and PSMA1 shRNAs, and protein total ubiquitin is detected by western blot. G) MDA‐MB‐231 cells were transfected with UBB, UBC, and PSMA1 shRNAs, respectively, then cisplatin treatment for 48 h, and cell viability is detected by ATP release assay. All values are presented as mean value (three replications) ± SD, *p < 0.05 and **p < 0.01 (C and E two‐tailed Student's *t*‐test).

To identify the most effective alterations that are responsible for cisplatin resistance, we administrated cisplatin either alone or in combination with a whole‐genome RNA interference (RNAi) library containing 64 755 siRNAs for 21 585 genes in MDA‐MB‐231 cells (Figure S1A,B, Supporting Information). The *Z*‐score for each targeted gene was calculated and ranked to select candidate genes that modulate cisplatin sensitivity. Candidate genes with *Z*‐scores ≧2.5 were defined as sensitive genes (*n* = 45), whose knockdown contributes to cisplatin sensitivity, whereas genes with *Z*‐scores ≦ −2.5 (*n* = 104) were defined as resistant genes (**Figure** **1**A). We first performed a pathway enrichment analysis of all genes presented in the sensitive list and found that the top pathway was the ubiquitin proteasome system (UPS), which contains 16 candidate genes (Figure 1B and Table S1, Supporting Information).

The proteasome has important roles in cellular protein homeostasis, through which protein aggregates or damaged proteins are labeled with ubiquitin for degradation. Thus, we tested the total protein ubiquitination levels after cisplatin administration, and the data revealed that cisplatin treatment caused an increase in protein ubiquitination in a time‐dependent manner (Figure 1C). The remarkably accumulated protein ubiquitination suggests that cisplatin treatment may cause protein damage. Next, we attenuated the UPS by targeting the top three candidate genes from the sensitive list (PSMC6, PSMD8, and PSMA1, Figure 1D) to block the degradation of damaged proteins. After depletion of the proteasome, cisplatin‐induced cell apoptosis was dramatically increased (Figure 1E), which is associated with accumulation of damaged proteins in the combined treatment group (Figure 1F). The cellular ubiquitin comes from the ubiquitin pool, which is consisted of Ubiquitin B (UBB) and Ubiquitin C (UBC) that is also a top hit among our cisplatin sensitive list. Thus, we blocked the total protein ubiquitin by targeting UBB or UBC using shRNAs, and the data revealed a markedly reduced cell number and increased apoptosis compared to cisplatin single treatment (Figure S1C,D, Supporting Information). In accordance with these results, the cellular ATP production was significantly decreased by shRNAs for UBB, UBC, and PSMA1 under the treatment of cisplatin (Figure 1G). All these data suggest that targeting the UPS system serves as an important strategy to enhance efficacy of cisplatin through accumulation of damaged protein.

### 2.2 Suppression of Genes Encoding Mitochondrial Respiration Complexes is Associated with Cisplatin Resistance

**Figure 2:**
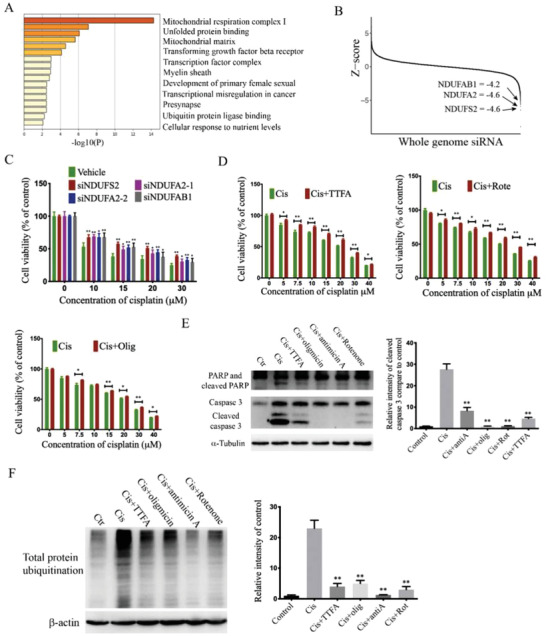
Genome‐wide RNAi screening identifies mitochondria as a target which contribute to cisplatin resistance. A) After RNAi screening, candidate genes with *Z* score ≦ −2.5 are sorted out as resistant list. 104 candidate genes were sorted out, and enrichment analysis of these genes shows the mitochondrial respiration complex I. B) The whole genome siRNAs were ranked by *Z*‐score and three top resistant candidates, NDUFAB1, NDUFA2, and NDUFS2, are laid out. C) siRNAs were designed for NDUFAB1, NDUFA2, and NDUFS2, cells were transfected with siRNAs and then treated with different concentration of cisplatin, cell viability is detected by Alamar Blue assay. D) MDA‐MB‐231 cells were treated with cisplatin alone or combined with nontoxic concentration of rotenone (20 × 10^−9^ m), TTFA (2 × 10^−6^ m), and oligomycin (1 × 10^−9^ m), respectively, and cell viability is detected by Alamar Blue assay. E) MDA‐MB‐231 cells were treated with indicated drugs for 48 h, and cleaved caspase 3 and PARP were detected by western blot. F) MDA‐MB‐231 cells were treated with cisplatin alone or combined with mitochondrial inhibitors, and protein total ubiquitin was detected by western blot and gray value was measured for each group of three independent assays. All values are presented as mean value (three replications) ± SD, *p < 0.05 and **p < 0.01. (C–F, two‐tailed Student's *t*‐test)

Meanwhile, our studies on the cisplatin resistant list (Figure 1A), which contains 104 candidate genes, revealed that the mitochondrial respiration complex I and mitochondrial matrix were ranked as the first and third effected cell components, respectively (**Figure** **2**A and Table S2, Supporting Information). These results implied that compromised mitochondrial activity mediated by siRNA knockdown might enhance cisplatin resistance. To investigate this possibility, three top candidate genes, NDUFS2, NDUFA2, and NDUFAB1, which are components of the mitochondrial respiration complex I and have the lowest *Z*‐scores (−4.6, −4.6, and −4.2, respectively) (Figure 2B), were chosen to validate the RNAi screening results. Our data indicated that after knockdown of these genes by siRNAs, the cells showed higher cell viability upon cisplatin treatment compared to the siRNA vehicle group (Figure 2C). To further validate these results, we also tested effects of three inhibitors (rotenone, thenoyltrifluoroacetone (TTFA), and oligomycin), which target for mitochondrial complexes I, II, and V, respectively, and found they all elicited protective effects for MDA‐MB‐231 cells against cisplatin‐induced killing (Figure 2D). To further investigate the relationship of mitochondrial respiration chain and cisplatin resistance, we assessed cell apoptosis after cisplatin treatment alone or in combination with mitochondrial complex inhibitors. Consistent with the cell viability assay, the respiration chain inhibitors could all suppress cisplatin‐induced cleavage of poly(ADP‐ribose) polymerase (PARP) and caspase 3 (Figure 2E).

Our earlier study on the sensitive list demonstrated that cisplatin induces protein ubiquitination, which is responsible for cell death. To investigate the potential effect of mitochondrial complex I, II, and V on ubiquitination, we co‐treated MDA‐MB‐231 cells with cisplatin and their respective inhibitors. Our data indicated that all the inhibitors blocked protein ubiquitination induced by cisplatin (Figure 2F). Thus, our genome‐wide siRNA screening uncovered that cytotoxic effect of cisplatin is enhanced by disruption of the UPS and was minimized by downregulation of mitochondrial respiration complexes. Although protein ubiquitination was the intersection point, the underlying mechanism for downregulation of mitochondrial respiration complex affects ubiquitination is unclear.

### 2.3 An Evolution Model Identifies Graded Reduction of Mitochondrial Function and Enhanced Protein Damage Clearance is Responsible for Cisplatin Resistance

**Figure 3:**
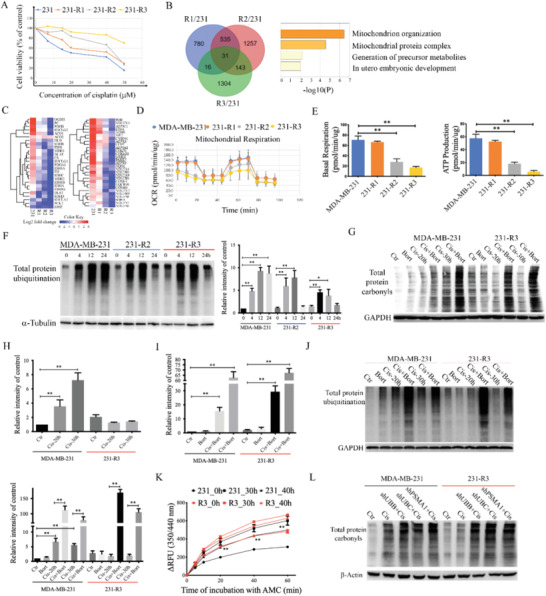
An evolution model identifies graded reduction of mitochondrial function and enhanced protein clearance is responsible for cisplatin resistance. A) Cell viability under the treatment of different concentration of cisplatin was detected by Alamar Blue assay in MDA‐MB‐231, 231‐R1, 231‐R2, and 231‐R3 cells. B) 31 genes were identified by Venn diagram comparing expression of genes that are downregulated in the 231‐R1, 231‐R2, and 231‐R3 cell lines compared to parental MDA‐MB‐231 cells. Enrichment analysis of the 31 genes showed that mitochondrial organization and mitochondrial protein complex are associated with cisplatin resistance. C) Genes from the citric acid cycle and mitochondria respiratory chain are gradually down‐regulated in the cisplatin resistant cell lines. D) Mitochondrial bioenergetic capacity of MDA‐MB‐231, 231‐R1, 231‐R2, and 231‐R3 cells are detected via Seahorse XFp Cell Mito Stress Test assays, and E) basal respiratory capacity and ATP production of cisplatin resistant cells were dramatically decreased compared with the parental MDA‐MB‐231 cells. F) MDA‐MB‐231, 231‐R2, and 231‐R3 cells were treated with cisplatin as indicated time, and protein total ubiquitin was detected by western blot and gray value was measured for each group of three independent assays. G) MDA‐MB‐231 and 231‐R3 cells were treated with cisplatin alone or combined with bortezomib, and protein total carbonyls were detected by DNPH (2,4‐dinitrophenylhydrazine) labeling and western blot and gray value was measured for H) cisplatin treatment alone, and I) combined treatment with bortezomib of three independent assays. J) MDA‐MB‐231 and 231‐R3 cells were treated with cisplatin alone or combined with bortezomib treatment, and protein total ubiquitin was detected by western blot and gray value was measured for each group of three independent assays. K) Effects of cisplatin treatment on the proteasome activity. MDA‐MB‐231 and 231‐R3 cells were treated with cisplatin (5 × 10^−6^ m) as indicated time, and the proteasome activity is measure by fluorescent AMC tagged peptide substrate. L) MDA‐MB‐231 and 231‐R3 cells were treated with cisplatin alone or combined with shRNAs for UBB, UBC, and PSMA1, respectively, and protein total ubiquitin was detected by western blot. All values are presented as mean value (three replications) ± SD, *p < 0.05 and **p < 0.01. (E–J, two‐tailed Student's *t*‐test; K, one‐way ANOVA with Bonferroni's post‐test.)

On the other front, our efforts to establish acquired resistance through an evolution process by gradually increasing concentrations of cisplatin obtained three resistant cell lines with stable tolerance to cisplatin at 1 × 10^−6^ m (231‐R1), 3.5 × 10^−6^ m (231‐R2), and 10 × 10^−6^ m (231‐R3) (Figure S2A, Supporting Information). In a test of increasing concentrations of cisplatin, the 231‐R3 cells were highly resistant even when the concentration was increased to 50 × 10^−6^ m, while the 231‐R1 and 231‐R2 cells exhibited partial resistance (**Figure** **3**A).

Because our initial effort using 15 × 10^−6^ m cisplatin failed to obtain any resistant cells, we believed the initial low dose of cisplatin selectively enriched cells with tolerable changes, and every consequent screening with graded increased drug stress might select and enrich cells with further tolerable changes, which eventually enabled the cells to become fully resistant (Figure S2B, Supporting Information). To identify these changes, we conducted RNA‐Seq to these three cell lines and the parental MBA‐MD‐231 cells. We detected 31 genes that were shared in all three resistant cell lines (Figure 3B), and most of them play a role in mitochondrion‐related function: tricarboxylic acid cycle (TAC cycle) and mitochondrial respiration complex. Of note, many of the mitochondrial‐related genes displayed a graded reduction in their expression from MDA‐MB‐231, 231‐R1, 231‐R2 to 231‐R3 cells (Figure 3C). Consistent with the dramatical decrease in mitochondrial gene expression, the mitochondrial respiration capacity displayed a graded decrease in these cisplatin resistant cell lines (Figure 3D). Similar decreases in mitochondrial basal respiratory capacity and ATP production were also observed (Figure 3E). Thus, these data reveal that the gradient reduction in mitochondrial respiratory activity is correlated with gradually increased resistance cells established during the prolonged cisplatin stressing.

Because our RNAi screening result identified that the proteasome was essential in tackling cisplatin assaults, we examined the ubiquitination and proteasome activities in these cells. The data indicated the cisplatin‐induced protein ubiquitination at comparable levels in the MDA‐MB‐231, 231‐R2, and 231‐R3 cells 4 h after the exposure. While the ubiquitination reached a high level in MDA‐MB‐231 cells at 12 h and maintained high at 24 h, it was slightly or not increased in 231‐R2 and 231‐R3 cells at 12 h and returned to basal levels at 24 h (Figure 3F). High levels of ubiquitination are usually triggered by protein damage. To verify this, we examined protein carbonyls, which are irreversible and unrepairable oxidative protein damage and are marked for proteolysis by the proteasome.^[^ ^13^, ^14^ ^]^ We found that cisplatin treatment led to accumulated protein carbonyls in the MDA‐MB‐231 cells in a timely manner, while such an effect was not observed in the 231‐R3 cells (Figure 3G,H). To confirm this is indeed caused by the high proteasome activity in the 231‐R3 cells, we did the following two experiments. First, we treated cells with bortezomib, a potent proteasome inhibitor, and the data indicated that bortezomib increased protein damage in both MDA‐MB‐231 and 231‐R3 cells, and the level of protein damage in the 231‐R3 cells was slightly higher than the MDA‐MD‐231 cells (Figure 3G,I). Consistently, the protein ubiquitination exhibited similar pattern with the accumulation of oxidative damaged protein (Figure 3J). Then, we conducted a proteasome activity assay and found it was induced by cisplatin in both MDA‐MB‐231 and 231‐R3 cells, with much stronger cisplatin resistant cells (Figure 3K). On the other hand, we also attenuated the UPS system by treatment with shRNAs for UBB, UBC, and PSMA1, respectively, and protein damage induced by cisplatin treatment was increased by these shRNAs (Figure 3L). These results indicate that the mitochondrion respiratory system‐dependent efficacy is reversed in 231‐R3 cells by shRNAs for UBB, UBC, and PSMA1, which recaptured the effect of bortezomib treatment. Besides bortezomib treatment, two other proteasome inhibitors (MG132 and carfilzomib) also increased protein damage in both MDA‐MB‐231 and 231‐R3 cells (Figure S2C, Supporting Information). Consistent with the enhanced protein damage, ATP production in combined treatment group is decreased (Figure S2D, Supporting Information) and cell death in combined treatment group is increased (Figure S2E, Supporting Information).

Thus, our unbiased genome‐wide siRNA screening and evolutionary cisplatin stress model reveal that cisplatin resistance is caused by accelerated protein damage clearance, which is inhibited by mitochondrial respiratory activity and enhanced by proteasome activity.

### 2.4 Enhancement of Damaged Protein Clearance Causes Cisplatin Resistance through Blocking Mitochondrial Dynamics and Caspase Activation

**Figure 4:**
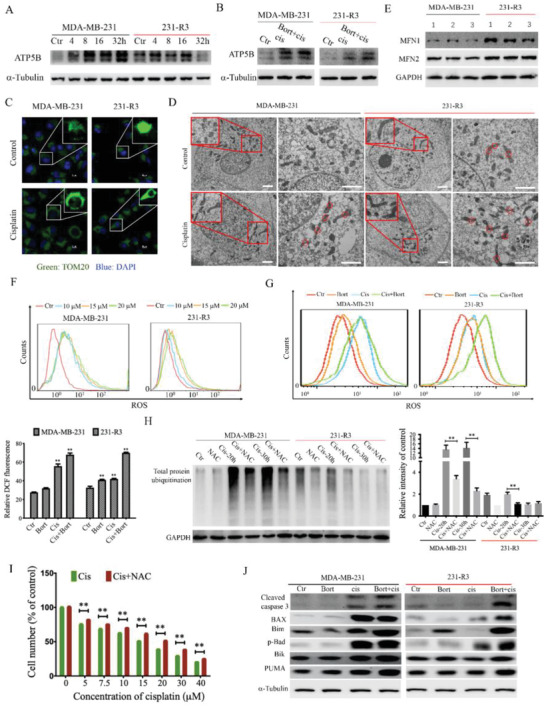
Enhancement of damaged protein clearance causes cisplatin resistance through blocking mitochondrial dynamics and caspase activation. A) MDA‐MB‐231 cells and 231‐R3 cells were treated with cisplatin as indicated time, and pre‐ATP5B (upper bind) and mature ATP5B (lower bind) were detected by western blot analysis. B) MDA‐MB‐231 and 231‐R3 cells were treated with indicated drugs for 48 h, and pre‐ATP5B were detected by western blot analysis. C) MDA‐MB‐231 and 231‐R3 cells were treated with cisplatin for 12 h, mitochondrial is visualized by immunofluorescence staining of TOM20. D) Electron microscopy images of MDA‐MB‐231 and 231‐R3 cells which are treated with cisplatin, and nodes of mitochondrial fusion are indicated by circles, Scale bar: 2 µm. E) Basal MFN1 and MFN2 expression in MDA‐MB‐231 and 231‐R3 cells were detected by western blot. F) MDA‐MB‐231 and 231‐R3 cells were treated with cisplatin as indicated concentration, and cells were stained with DCFH‐DA and analyzed for fluorescence by flow cytometry. G) MDA‐MB‐231 and 231‐R3 cells were treated with indicated drugs, and cells were stained with DCFH‐DA and analyzed for fluorescence by flow cytometry. H) MDA‐MB‐231 and 231‐R3 cells were treated with cisplatin alone or combined with NAC treatment, and protein total ubiquitin was detected by western blot and gray value was measured for each group of three independent assays. I) MDA‐MB‐231 cells are treated with cisplatin alone or combined with NCA, and cell viability is detected by Alamar Blue assay. J) MDA‐MB‐231 and 231‐R3 cells were treated with cisplatin or combined with bortezomib treatment, and cleaved caspase 3 and pro‐apoptosis Bcl2 family members were detected by western blot analysis. All values are presented as mean value (three replications) ± SD, *p < 0.05 and **p < 0.01. (G–I, two‐tailed Student's *t*‐test.)

Next, we investigated how mitochondrial respiratory activity affects protein damage clearance and vice versa. It is known that majority mitochondrial proteins are synthesized in the cytoplasm and imported into the mitochondrion after proper processing.^[^ ^15^ ^]^ We suspected that the protein damage caused by cisplatin might affect this process. To investigate this possibility, we examined the status of the ATP synthase F1 subunit beta (ATP5B), which is synthesized in the cytoplasm and transported to the mitochondria where it matures and serves as an important aspect of cellular protein homeostasis.^[^ ^16^ ^]^ Consistent with the total protein ubiquitination results, the pre‐ATP5B was dramatically accumulated by cisplatin treatment in MDA‐MB‐231, whereas the accumulation was significantly less in 231‐R3 and disappeared at 32 h (**Figure** **4**A). To verify that the faster disappearance of pre‐ATP5B in 231‐R3 cells was associated with higher proteasome activity, we treated the cells with bortezomib, and the data indicated that the bortezomib treatment increased pre‐ATP5B (Figure 4B). ATP5B plays an important role in mitochondrial dynamics, i.e., mitochondrial fission and fusion, which regulate mitochondrial homeostasis and quality.^[^ ^17^, ^18^, ^19^ ^]^ Thus, we visualized mitochondrial morphology in 231‐R3 and MDA‐MB‐231 cells by TOM20 (a marker of the mitochondrial outer membrane protein) immunofluorescence staining and found that 231‐R3 cells exhibited more extensive mitochondrial fusion than did the MDA‐MB‐231 cells, and enhanced mitochondrial fusion was also observed in both cell lines upon cisplatin treatment (Figure 4C). This phenomenon was confirmed by visualizing with the electron microscope (Figure 4D). Consistent with this phenotype, increased expression of mitofusin 1 and 2 (MFN1/2), which promote mitochondrial fusion, was observed in 231‐R3 cells compared with MDA‐MB‐231 cells (Figure 4E). Upon cisplatin treatment, MFN1 expression was increased in both cell lines, meanwhile, the cisplatin did not affect expression of dynamin‐related protein 1 (DRP1) that facilitates mitochondrion fission, although its level was lower in 231‐R3 cells than MDA‐MB‐231 cells (Figure S2F, Supporting Information). All these results suggest that protein damage caused by cisplatin can impair mitochondrial dynamics and reduce its energetic capacity.

Mitochondrial damage is known to promote reactive oxygen species (ROS) generation.^[^ ^20^ ^]^ To examine this, we monitored ROS production in the MDA‐MB‐231 and 231‐R3 cells, and the data indicated that both cells displayed similar basal level of ROS; however, cisplatin at 10 × 10^−6^, 15 × 10^−6^, and 20 × 10^−6^ m markedly increased ROS in the MDA‐MB‐231 cells, whereas their induction was much milder in the 231‐R3 cells (Figure 4F). The presence of bortezomib completely abrogated the resistance of 231‐R3 to ROS induction (Figure 4G), suggesting that higher proteasome activity in 231‐R3 cells protects mitochondrion from cisplatin‐induced damage. In support of this hypothesis, we found that protein ubiquitination in the MDA‐MB‐231 cells caused by cisplatin treatment was significantly attenuated by *N*‐acetyl‐L‐cysteine (NAC), which blocks ROS production, and similar effect was also observed in 231‐R3 cells, although they originally had little ROS accumulation (Figure 4H). Consistently, blocking ROS production by NAC in MDA‐MB‐231 cells significantly attenuated the killing effect of cisplatin at all doses tested (Figure 4I). These results indicate that cisplatin induced much less mitochondrial damage in the cisplatin‐resistant 231‐R3 cells because of their high levels of proteasome activity, as bortezomib treatment completely abrogated this protective effect.

Caspase inactivation is frequently involved in drug resistance. Next, we investigated whether the reduced caspase activation in the 231‐R3 cells was due to their increased protein damage clearance. We found that cisplatin at 20 × 10^−6^ m, which induced caspase 3 activation in the MDA‐MB‐231 cells (Figure 4J), failed to induce obvious caspase 3 activation in the 231‐R3 cells (Figure 4J). We then treated cells combined with bortezomib and found that the addition of bortezomib activated caspase 3 in both MDA‐MB‐231 and 231‐R3 cells (Figure 4J). In accordance with the caspase 3 activation, multiple pro‐apoptosis Bcl2 family members, which are inactivated in 231‐R3 cells, were significantly induced by combination with bortezomib treatment (Figure 4J). Altogether, our data indicate that while protein damage caused by cisplatin activated caspase to kill cells, it also inhibits mitochondrial functions, possibly by activating the mitochondrial protein import surveillance system. This feedback loop between the mitochondria and protein damage results in the activation of proteasome to enhance protein damage clearance and allow cells to survive from drug stress.

### 2.5 Enhanced Proteolysis Serves as a Common Resistance Mechanism

Next, we investigated whether this finding could be applicable to other breast cancer cell lines. Our data demonstrated that the combination of cisplatin and bortezomib greatly enhanced the killing effect compared with a single cisplatin treatment in all the cell lines tested (MDA‐MB‐231, 231‐R1, 231‐R2, and 231‐R3) and two other different breast cancer cell lines (MCF7 and TM91). Despite their differential responses to cisplatin monotreatment, marked synergy was observed in all cell lines (Figure S3, Supporting Information). This observation indicates that inhibition of proteasome is a good strategy for enhancing efficacy of cisplatin for them.

**Figure 5:**
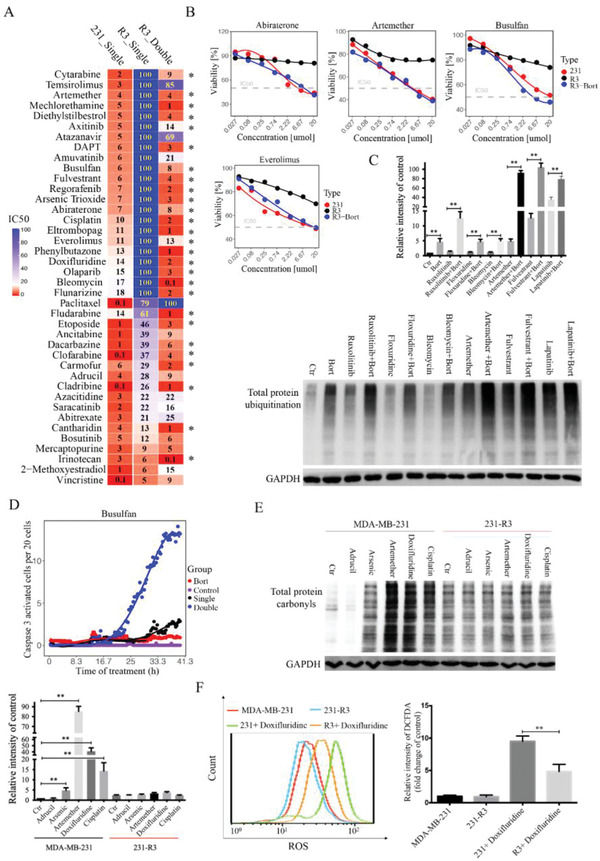
Proteasome activity serves as common resistant mechanisms for many anticancer drugs. A) 69 drugs’ screening identified 40 resistant drugs (231‐R3 to MDA‐MB‐231, fold change of IC50 > 5). Drug IC50 were shown for drug single treatment in MDA‐MB‐231 and 231‐R3 cells, and drug double treatment in 231‐R3 cells. Resistance to 27 drugs in 231‐R3 cells was reversed by bortezomib treatment (231‐R3_single to 231‐R3_double, fold change of IC50 > 5, asterisk). B) Resistance to abiraterone, artemether, busulfan, and everolimus in 231‐R3 cells was reversed by bortezomib treatment. C) MDA‐MB‐231 cells were treated with indicated drugs, and protein total ubiquitin was detected by western blot and gray value was measured for each group of three independent assays. D) FRET‐based caspase 3 (C3) biosensor‐labeled MDA‐MB‐231 cells were treated with busulfan or in combination with bortezomib treatment, and dynamic change of caspase 3 activation was monitored during 50 h treatment for each group. E) MDA‐MB‐231 and 231‐R3 cells were treated with indicated resistant drugs, and protein total carbonyls were detected by DNPH (2,4‐dinitrophenylhydrazine) labeling and western blot. F) MDA‐MB‐231 and 231‐R3 cells were treated with doxifluridine, and cells were stained with DCFH‐DA and analyzed for fluorescence by flow cytometry. All values are presented as mean value (three replications) ± SD, *p < 0.05 and **p < 0.01. (C, E, and F, two‐tailed Student's *t*‐test.)

We believe that cells selected from evolution model might carry changes that may induce cell tolerance to many other drugs. To test this supposition, we compared the 231‐R3 cells and the parental MDA‐MB‐231 cells using a drug library that contains 69 anticancer drugs approved by the Food and Drug Administrations (FDAs) of US or other countries (Table S3, Supporting Information). We found that 231‐R3 cells were significantly more resistant than MDA‐MB‐231 cells to 40 drugs, with a 2‐ to 100‐fold increase in the IC50 for the 231‐R3 cells (**Figure** **5**A). In the remaining drugs, three were more toxic to the 231‐R3 cells than to the MDA‐MB‐231 cells, and 26 showed no difference (Figure S4, Supporting Information). To test if the enhanced proteolysis activity plays a role in the resistance to these drugs, we treated the cells with the 40 drugs with bortezomib at the IC15 concentration. After combination with bortezomib treatment, the resistance to 27 out of 40 drugs was markedly reversed (Figure 5A,B and Figure S4, Supporting Information). We also tested protein homeostasis upon treatment with these drugs combined with bortezomib, and 6 of the 11 drugs tested showed high levels of protein ubiquitination after blockage of proteolysis activity (Figure 5C and Figure S5A, Supporting Information). These observations indicate that enhanced proteolysis is a common resistance mechanism for many anticancer drugs, and a blockage of protein degradation can overcome multidrug resistance in different cancer cells.

To further demonstrate the power of targeting proteasome activity for overcoming drug resistance, we monitored the dynamics of caspase 3 activation using a fluorescence resonance energy transfer (FRET)‐based caspase 3 (C3) biosensor system,^[^ ^21^ ^]^ which is a highly sensitive indicator of caspase 3 activation in intact living cells. The FRET ratio (yellow fluorescent protein (YFP) signaling divided by cyan fluorescent protein (CFP) signaling, Figure S5B, Supporting Information) gradually decreased upon the cleavage of caspase 3, which is associated with a gradual increase in CFP signaling (Figure S5C, Supporting Information). We tested three resistant drugs (eltrombopag, artemether, and busulfan) with bortezomib treatment and monitored the dynamics of caspase 3 activation during 50 h of treatment using MDA‐MB‐231‐C3 cells. All the single treatments, including that of bortezomib, exhibited less caspase 3 activation, in contrast to the dramatic activation of caspase 3 in the double treatment groups (Figure 5D and Figure S5D, Supporting Information). In accordance with caspase 3 activation, we found that combination with bortezomib treatment induced significant increase of Bim and phosphorylated Bad for many anticancer drugs (Figure S5E, Supporting Information), and propidium iodide (PI) staining for cell death was increased (Figure S5F, Supporting Information). These observations indicate that enhanced proteolysis is a common resistance mechanism for many anticancer drugs, and blockage of protein degradation can overcome multidrug resistance in different cancer cells by inducing caspase activation.

In our earlier data, we found that reduced protein damage and ROS accumulation contributed to cisplatin resistance observed in 231‐R3 cells (Figure 4F,H). Then, we further tested five resistant drugs for their effects on protein damage and ROS accumulation, and the data revealed that all these drugs induced protein carbonyl content in the MDA‐MB‐231 cells were much higher than that in the 231‐R3 cells (Figure 5E). Next, doxifluridine was chosen to test ROS generation. Results indicate that doxifluridine, which had no effect on protein carbonyl content in 231‐R3 cells, induced less ROS generation in the 231‐R3 cells than it did in the MDA‐MB‐231 cells (Figure 5F). All these results indicated that reduced ROS accumulation contributed to protein homeostasis and enhanced resistance to many anticancer drugs.

### 2.6 Overcoming Drug Resistance in Colon and Breast Cancer Patients by Inhibiting Proteasome Activity

**Figure 6:**
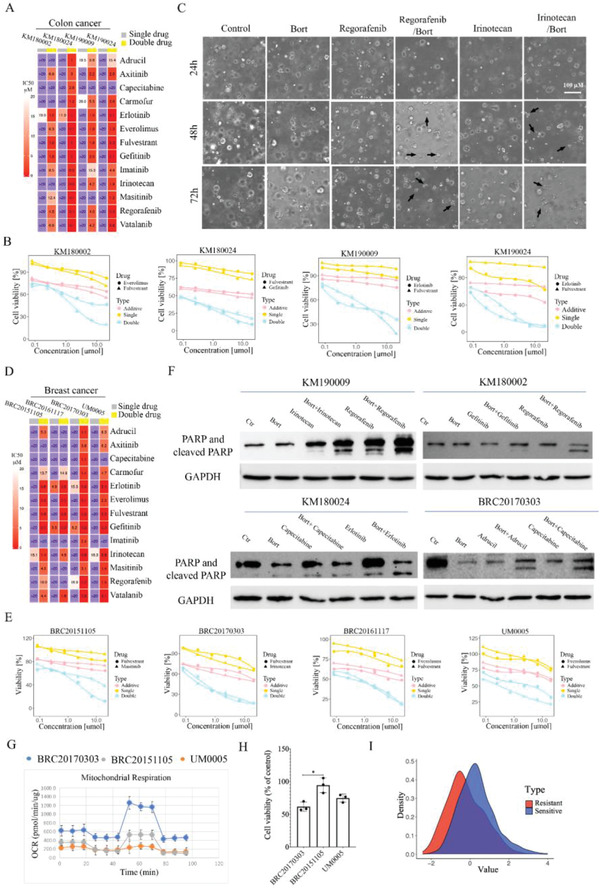
Overcoming multidrug resistance by targeting common mechanism in breast and colon cancer patients. A) 13 resistant drugs were tested in combination with bortezomib treatment in four colon cancer patients, and IC50 of 13 resistant drugs were shown for single drug treatment and double treatment, respectively. B) Two most effective synergy drugs for each colon cancer patient were demonstrated. Additive curves were calculated by Additive = E1 + E2 − E1*E2, where E1 is inhibition effect from single drug and E2 is inhibition effect from bortezomib treatment. C) Colon cancer patient sample KM190009 was treated with indicated drugs, and typical dead spheres were shown in double treated group. D) 13 resistant drugs were tested in combination with bortezomib treatment in four breast cancer patients, and IC50 of 13 resistant drugs were shown for single drug treatment and double treatment, respectively. E) Two most effective synergy drugs for each breast cancer patient were demonstrated. F) Colon cancer and breast cancer patients derived organoids were treated with indicated drugs, and PARP and cleaved PARP were detected by western blot. G) Three breast cancer patient samples, BRC20170303, BRC20151105, and UM0005 were detected for mitochondrial bioenergetic capacity via Seahorse XFp Cell Mito Stress Test assays. H) Three breast cancer patient samples, BRC20170303, BRC20151105, and UM0005 were treated with 20 × 10^−6^ m cisplatin for 48 h, and cell viability is detected by ATP production assay. I) The distribution of gene expression related to mitochondrial respiratory complex genes (*n* = 54) in breast cancer patients (*n* = 24) between resistant and sensitive group. All values are presented as mean value (three replications) ± SD, *p < 0.05 and **p < 0.01. (H, two‐tailed Student's *t*‐test.)

Because resistance to anticancer drugs is a major problem in cancer therapy, we next investigated whether our new finding could be used to overcome drug resistance in human cancers. Patient‐derived organoids (PDOs) has been shown to be reliable systems for cancer drug discovery and assessment.^[^ ^22^ ^]^ We have established culture conditions for PDOs from various cancers, including human breast and colon cancers. In the case of colon cancer, we found that four PDOs (KM180002, KM180024, KM190009, and KM190024) were quite resistant to many FDA‐approved drugs for colorectal cancer (red color) and some other tyrosine kinase inhibitors (Figure S6A, Supporting Information). Thus, we selected 13 drugs that are commonly resistant to these four PDOs and tested whether bortezomib treatment could be used to overcome their resistance. Results showed that effect of the single treatment was increased by bortezomib treatment (Figure S6B, Supporting Information), with a marked reduction in IC50 (**Figure** **6**A). We also conducted synergy analysis for these 13 drugs in the four colon cancer patients, and the data indicated that five drugs, four drugs, seven drugs, and seven drugs reached synergistic levels with bortezomib treatment in these four patients, respectively (Figure 6B and Figure S6C, Supporting Information).

The KM190009 colon cancer patient was quite malignant and had no response to multiple anticancer drugs, including regorafenib and irinotecan (Figure S6A, Supporting Information). After treatment with regorafenib and irinotecan, the KM190009 tumor grew spheres of similar size as in the control group, but tumor spheres were smaller at 48 h and became dead at 72 h in the double treatment with bortezomib (Figure 6C). Notably, bortezomib single treatment for 72 h had minor effect on spheres’ size compared to the control group, and only combined treatment group exhibited tumor killing effect (Figure 6C).

Next, we conducted the same test on four human breast cancer PDOs, which were found to be resistant to multiple drugs. Bortezomib treatment could also overcome their resistance, with dramatical decrease of cell viability (Figure S7A, Supporting Information) and IC50 (Figure 6D). Drug synergy analysis showed five drugs, two drugs, six drugs, and five drugs reached synergistic level with bortezomib treatment in these four patients, respectively (Figure 6E and Figure S7B, Supporting Information). Then tumor spheres were monitored in a breast cancer patient sample (BRC20170303) during 72 h treatment of different drug combinations. Notably, single treatment, including bortezomib, for 72 h had a minor effect on spheres’ size compared to the control group, and only the combined treatment groups exhibited a tumor killing effect (Figure S7C, Supporting Information).

After drug treatment for KM180002, KM180024, KM190009, and BRC20170303, proteins of these samples were further collected, and we found that the level of cleaved PARP was much higher in the double treated groups than it was in the drug‐resistant groups (Figure 6F). These results further indicated that enhanced proteolysis could serves as a general mechanism of drug resistance, and via combination with bortezomib treatment could overcome the resistance of many drugs for multitype of cancers.

To detect whether reduction of mitochondrial respiratory activity could also be detected in clinical breast cancer patients. We further investigated mitochondrial respiratory activity in three breast cancer patient‐derived organoids, and their response to cisplatin treatment. The results indicated that the organoid derived from BRC20170303 patient had the highest mitochondrial respiratory activity (Figure 6G), which was also the most sensitive one in response to cisplatin treatment (Figure 6H). The other two PDOs had lower mitochondrial respiratory activity, which are more resistance to cisplatin compared with BRC20170303 (Figure 6G,H). We also checked the expression of mitochondrial respiration complex genes (*n* = 54) in a cohort of breast cancer patients (GEO dataset, GSE6434, *n* = 24), which have sensitive and resistant groups. The distribution of gene expression related to mitochondrial respiration complex in the resistant group is lower than the sensitive group (Figure 6I). Of note, we found a significant decreased expression of mitochondrial complex I genes (NDUFA6, NDUFA7, NDUFS6, NDUFS7, and NDUFV1) in the resistant group (Figure S7D, Supporting Information), which is consistent with the RNAi screening results. The expression of these genes was moderately correlated in both the sensitive and resistant group (Figure S7E, Supporting Information). All these results indicate that reduction of mitochondrial respiratory activity could also be detected in clinical breast cancer patients.

### 2.7 Combined Treatment with Bortezomib to Overcome Cisplatin Resistance In Vivo and Alleviate Side Effects by Nanomicrosphere‐Packaged Drug Delivery

**Figure 7:**
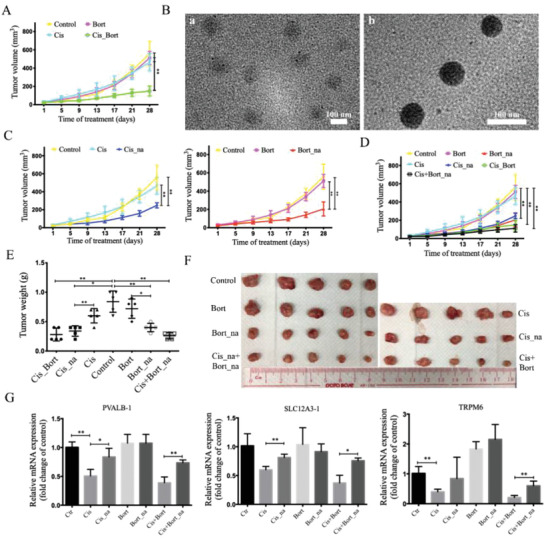
Alleviate side effect by PLA and PLGA microspheres‐packaged drug delivery. A) Nude mice bearing cisplatin resistant cells were treated with vehicle, cisplatin (5 mg kg^−1^), bortezomib (1 mg kg^−1^) for 28 days and tumor volumes were measured. Data are presented as the mean of five tumors for each group ± SD. B) TEM images of cisplatin and bortezomib‐loaded mPEG‐PLGA nanoparticles. a) Cisplatin‐loaded mPEG‐PLGA nanoparticles; b) bortezomib‐loaded mPEG‐PLGA nanoparticles. All images share the same scale bar, 100 nm. C,D) Nude mice bearing cisplatin resistant cells were treated with vehicle, cisplatin (5 mg kg^−1^), Cis_na (5 mg kg^−1^), bortezomib (1 mg kg^−1^), or Bort_na (1 mg kg^−1^) for 28 days and tumor volumes were measured. Data are presented as the mean of five tumors for each group ± SD. E) Tumor weight of seven groups is analyzed by *t*‐test. F) Tumor photograph of seven groups. G) q‐PCR assay of nephrotoxicity‐related genes in seven groups. All values are presented as mean value (three replications) ± SD, *p < 0.05 and **p < 0.01. (A, C, and D, one‐way ANOVA with Bonferroni's post‐test; E and G, two‐tailed Student's *t*‐test.)

Next, we investigated in vivo combination of cisplatin and bortezomib in a xenograft mouse model using cisplatin‐resistant cells. The results indicate that cisplatin or bortezomib single treatment virtually has no effect on inhibition of resistant tumor growth. While combined treatment of cisplatin and bortezomib synergistically inhibited tumor growth (**Figure** **7**A). However, we noted that side effects of combined treatment were also high, as indicated by drop of mouse body weight. To improve therapeutic responses and reduce side effects, we successfully developed bortezomib‐ or cisplatin‐loaded poly(ethyleneglycol)‐poly(lactic‐*co*‐glycolic acid) based nanoparticles (PEG‐PLGA NPs; named, Bort_na and Cis_na) to deliver an optimum dose of bortezomib and cisplatin for improved chemotherapy. Transmission electron microscopy (TEM) imaging revealed that the average size of spherical cisplatin‐loaded mPEG‐PLGA nanoparticles (Cis_na) and bortezomib loaded mPEG‐PLGA nanoparticles (Bort_na) is about 58.81 ± 4.5 and 106.09 ± 8.9 nm (Figure 7B), respectively, which was suitable for passive tumor targeting and accumulation in tumors through an enhanced permeability and retention effect.^[^ ^23^ ^]^ Free cisplatin had no obvious effect on the inhibition of resistant tumors, whereas the tumors were inhibited from the group treated with Cis_na compared to control group (*p* < 0.01) and free drug (*p* < 0.01) (Figure 7C). Similarly, the tumor inhibition from the group treated with Bort_na was enhanced compared to control group (*p* < 0.01) and free drug (*p* < 0.01) (Figure 7C). More importantly, mice treated with Bort_na combined with Cis_na achieved the strongest inhibition effects on tumor growth among the groups treated with single Bort_na, Cis_na, Cis_Bort, and free drugs (Figure 7D). The variation trend of tumor volume was consistent with that of tumor weight and tumor photographs (Figure 7E,F), where the Bort_na combined with Cis_na led to the largest reduction in tumor weight. As biocompatible PEGylated PLGA NPs dramatically prolong their blood circulation by reducing nanoparticle uptake by non‐Kupffer cells in liver, therefore resulting in high accumulation of drugs in tumor^[^ ^24^ ^]^ and decreasing the off‐target toxicity. To investigate the safety of different treatments, a quantitative polymerase chain reaction (q‐PCR) assay to detect renal distal convoluted tubule injury related genes was performed, which has been reported to be suppressed by cisplatin treatment.^[^ ^25^ ^]^ The results showed that nephrotoxicity, which was observed in free cisplatin treatment, was decreased in PEGylated cisplatin group (Figure 7G). In the co‐delivery of bortezomib‐ and cisplatin‐loaded PEG‐PLGA NPs, the nephrotoxicity was also decreased compared to the combined treatment of free bortezomib and cisplatin (Figure 7G). Consequently, this distinctive combined treatment strategy resulted in the prominent suppression of tumor growth, the attenuation of drug resistance and fewer side effects.

## 3 Discussion

Drug resistance is very common and occurs in virtually all types of cancers and all therapeutic agents, of which cisplatin resistance is most well‐known and extensively studied^[^ ^6^ ^]^ (Table S4, Supporting Information). Thus, we have attempted to identify genes whose alteration could overcome cisplatin resistance using a whole genome‐wide RNAi screening and an evolutionary drug stressing model. Both approaches revealed that enhanced protein clearance that shuts down caspase activation is responsible for cisplatin resistance. We further showed that this could serve as a common mechanism for cells to resist for many other anticancer drugs. Using drug‐resistant cells and PDOs derived from breast cancers and colon cancers, we found that this common drug‐resistance mechanism could be blocked by a combination of many anticancer drugs with bortezomib to activate caspase and to achieve effective therapy.

Cancer cells usually adapt multiple strategies to counteract the treatment of an anticancer drug in order to survive and eventually acquire resistance to the drugs.^[^ ^1^ ^]^ Using a library containing siRNAs for 56 genes to enhance cisplatin sensitivity of MDA‐MB‐231 cells, we have previously identified ATP7A as a top synthetic lethal gene that sequesters cisplatin and pumps it outside the cell, preventing it from entering the nucleus to bind to its primary target, DNA.^[^ ^10^ ^]^ This mechanism of resistance belongs to pre‐target mechanism. Using a library containing siRNAs for 704 kinases, we demonstrated that cisplatin, while damaging DNA, concomitantly activates ATR‐CHK1‐WEE1 signaling, which holds cells in the S‐phase for repairing their damaged DNA before releasing them into cell cycle.^[^ ^11^ ^]^ This mechanism, which attenuates cisplatin‐induced lethality, represents an example of on‐target mechanism. The current genome‐wide screening, as it includes nearly all the genes, in theory, could identify many mechanisms for cisplatin resistance, which is reflected by alterations of many signaling pathways revealed by our pathway analyses on the genes whose knockdown renders cisplatin sensitivity or cisplatin resistance. However, since we focused on genes whose knockdown caused highest sensitivity or resistance, we identified genes that encode proteasome as the sensitive genes, whose knockdown rendered cells higher sensitivity to cisplatin, and genes encoding mitochondrial respiratory complex were resistant genes, whose knockdown rendered cells higher resistance to cisplatin. Consistent with this finding, cisplatin‐resistant cells selected by an evolution drug pressure have higher proteasome activity and lower mitochondrial respiratory activity.

Several previous studies have shown that the toxicity of cisplatin could be synergized by bortezomib to induce cellular apoptosis under certain experimental conditions.^[^ ^26^, ^27^ ^]^ It was shown that bortezomib and cisplatin significantly increased Bim and Bik upregulation and activation of caspase 3.^[^ ^26^ ^]^ We found that the combination of bortezomib and cisplatin strongly induced caspase 3 activation, apoptosis, and upregulation of several proteins involved in apoptosis, including Bim and Bik, in cisplatin‐resistant 231‐R3 cells, whereas in the parental MDA‐MB‐231 cells, cisplatin alone could achieve the similar effect. It was also shown that bortezomib prevents cisplatin‐mediated degradation of CTR1, which plays an important role in transportation of cisplatin before it hit its cellular targets, and effectively increases platinum accumulation in intraperitoneal ovarian carcinomas, leading to the enhanced cytotoxicity.^[^ ^27^ ^]^ While multiple factors could potentially affect actions of cisplatin and bortezomib, we believe that the enhancement of proteolysis to clear damaged proteins to avoid caspase activation represents the most effective approach for host defense system adaptation and drug resistance, as evidenced by our finding that the toxicity of many (40/69) anticancer drugs tested is markedly enhanced by bortezomib.^[^ ^28^ ^]^ Thus, cells that adapt the enhanced proteolysis play important role in triggering broad drug resistance.

We demonstrated that many anticancer drugs, besides cisplatin, cause profound protein damage, especially when there is the blockage of proteasome activity by bortezomib. It is no doubt that the accumulation of damaged proteins will trigger apoptosis, leading to the drug sensitivity. The protein damaging, on the other hand, also targets the mitochondrion and impairs its function, which enables cells to gain drug resistance, as we found that treatment with inhibitors for respiratory complexes ellicts better cell viability by attenuating caspase activation. We believe that this action is primarily because 99% of mitochondrial proteins are made in the cytoplasm of the cell and imported into the mitochondrion.^[^ ^29^ ^]^ The damaged proteins, if transported into the mitochondria, will certainly cause mitochondrial damage and impair their function. Meanwhile, the damaged protein may also activate mitochondrial protein import surveillance checkpoint, leading to mitochondrial stress, which usually occurs when cells are treated with drugs, suffered virus infection, or other exogenous assaults.^[^ ^30^, ^31^ ^]^ Both the mechanisms can reduce the quantity of the functional mitochondria and diminish mitochondrial energy (ATP) production (Figure 3D,E), which may activate proteasome activity, as demonstrated previously,^[^ ^32^, ^33^ ^]^ to enhance the clearance of damaged protein and cell viability.

Of note, many anticancer drugs, which damage proteins, do not damage DNA, in contrast to cisplatin, which damages both protein and DNA. The underlying mechanisms for protein damaging of these drugs are unclear as many of them target different cellular components compared with cisplatin, such as arsenic trioxide, which targets thioredoxin reductase;^[^ ^34^ ^]^ capecitabine, which blocks DNA synthesis;^[^ ^35^ ^]^ erlotinib, which is a tyrosine kinase inhibitor for epidermal growth factor receptor (EGFR);^[^ ^36^ ^]^ and regorafenib, a multi‐kinase inhibitor targeting EGFR.^[^ ^37^ ^]^ Nonetheless, this critical finding allows us to further investigate the combination of bortezomib, which blocks the clearance of damaged proteins, with some of these drugs and enables us to demonstrate in principle that the strategy of targeting the common drug‐resistance mechanism may be an effective approach for cancer therapy.

In summary, we found that many anticancer drugs induce protein damage, which is a potent and also a common way to kill cells through activating caspase and apoptosis (Figure S8, Supporting Information). Meanwhile, the damaged proteins also have a profound effect on the mitochondria through activating the protein import surveillance checkpoint to block mitochondrial dynamics, impair mitochondrial respiratory activity, and reduce ATP production, leading to the activation of proteasome activity to enhance clearance of damaged proteins (Figure S8, Supporting Information). We further demonstrated that many anticancer drugs could trigger this type of feedback loop for the development of broad drug resistance. Finally, we showed that disruption of ubiquitination proteasome system by bortezomib treatment could enhance cytotoxic of many anticancer drugs both in established cancer cell lines and patient‐derived organoids. Thus, this study demonstrates a feasibility for eliminating broad drug resistance by targeting this common mechanism to achieve effective therapy for multiple cancers.

## 4 Experimental Section

##### Cell Lines and Cell Culture

The human cancer cell lines MDA‐MB‐231, MCF‐7, SUM 149, and TM91 were obtained from ATCC and cultured in Dulbecco's modified Eagle medium (DMEM, Gibco) supplemented with 10% fetal bovine serum (Gibco), 1 × 10^−3^ m L‐glutamine (Gibco), and 1% nonessential amino acids (Gibco). The cisplatin‐resistant breast cancer cell line 231‐R1, 231‐R2, and 231‐R3 were established by chronically exposing parental MDA‐MB‐231 cells to gradually increased concentrations of cisplatin (Sigma) starting from 0.1 to 3 µg mL^−1^ for over 1 year until they became resistant (Figure S2A, Supporting Information).

##### High‐Throughput RNAi Screen

The whole genome RNAi library screening was performed in National Institutes of Health (USA), and the procedure of the RNAi screening was reported in earlier study.^[^ ^11^, ^38^ ^]^ Briefly, MDA‐MB‐231 cells were planted in 384‐well plate (Corning 3570), 20 µL of serum‐free media containing Lipofectamine Max (ThermoFisher) and siRNA sequence was added to each well of the plates. Cells were further cultured for 24 h for favorable transfection efficiency, and cisplatin (10 × 10^−6^ m, ≈EC30 for MDA‐MB‐231 cells) or vehicle (10 µL DMEM) was added to each well of the plates and the cell viability was tested by Cell Titer Glo (Promega) after 72 h of cisplatin treatment. The whole genome RNAi screening was conducted using the Ambion Silencer Select Human whole genome Library. This library targeted 21 585 human genes with three individual siRNA sequences for each gene. To select candidate genes that modulate cisplatin activity, the *Z*‐score for each individual gene was calculated as: *z* = (*x* − *μ*)/*σ*, where *x* is the experimental value; *μ* is the median screen value; and *σ* is the standard deviation for the screen. Pathway enrichment analysis was performed based on KEGG pathway, GO biological processes, GO cellular components, and GO molecular functions.

##### 69 Drug Library Screening

MDA‐MB‐231 cells and 231‐R3 cells were planted in 384‐well plate, and the following day a seven‐point threefold dilution series of 69 drugs was dispensed into 384‐well microplates either alone or in combination with bortezomib treatment. After 48 h treatment, cell viability was measured by Cell Titer Glo 2.0 Luminescent assay.

##### Human Tissues

Colorectal samples were obtained from Kiang Wu Hospital. Breast samples were obtained from the University Hospital and Zhuhai People's Hospital with informed consent and the study was approved by the ethical committee. All patients were diagnosed with colorectal cancer or breast cancer. From the resected patient samples, tumor cells were isolated as described by Sato et al.^[^ ^39^ ^]^

##### Organoid Culture

Colon cancer organoids and breast cancer organoid were cultured in organoid medium as described by van de Wetering et al. and Sachs et al., respectively.^[^ ^40^, ^41^ ^]^

##### Organoid Viability Assays

10 µL of DMEM F12 medium containing 50% matrigel matrix bulk was dispensed into 384‐well microplates. Organoids were dissociated and trypsinized before being resuspended in organoid medium, and 1000 cells were dispensed into each well of 384‐well microplates. After 48 h culture, a six‐point threefold dilution series of each drug was dispensed into 384‐well microplates and cell viability was measured by Cell Titer Glo 2.0 Luminescent assay following 4 days of drug incubation.

##### Lentivector Short Hairpin (sh) RNA Transfection

The sequences of different shRNAs and their knockdown efficiency are listed in Table S5 in the Supporting Information. The shRNAs were cloned into the lentiviral vector pLKO.1 puro (Addgene Plasmid 10878) according to the manufacturer's instructions. After transfection, positive cells were selected by Puromycin (thermofisher), and knockdown effect of the shRNAs was evaluated by real‐time PCR using Taqman Gene Expression Master Mix (Life Technologies).

##### Chemicals and Antibodies

PARP antibody (Cell Signaling Technology), caspase 3 antibody (Cell Signaling Technology), GAPDH antibody (Santa Cruz Biotechnology), Anti‐Ubiquitin antibody (Sigma‐Aldrich), Anti‐ATP synthase antibody (merckmillipore), OPA1 antibody (Cell Signaling Technology), *α*‐Tubulin (Sigma‐Aldrich), CTR1 (PROTEINTECH), Pro‐Apoptosis Bcl‐2 Family Antibody Sampler Kit (Cell Signaling Technology) were used. All the inhibitors in this study were used at the indicated concentration unless stated independently: mitochondrial complex III inhibitor antimycin A (sigma A8674; 5 × 10^−6^ m), mitochondrial complex II inhibitor thenoyltrifluoroacetone (sigma T27006; 2 × 10^−6^ m), mitochondrial complex V inhibitor oligomycin (sigma 75351; 1 × 10^−6^ m), mitochondrial complex I inhibitor rotenone (sigma R8875; 5 × 10^−9^ m), cisplatin (sigma; 20 × 10^−6^ m), *N*‐acetyl‐L‐cysteine (sigma A7250; 5 × 10^−3^ m), MG132 (selleckchem S2619; 150 × 10^−9^ m), bortezomib (selleckchem S1013; 25 × 10^−9^ m), carfilzomib (selleckchem PR‐171; 10 × 10^−9^ m), FCCCP (sigma; 10 × 10^−6^ m). Corning matrigel matrix bulk (Corning), epidermal growth factor (Thermo), B27 supplement (Gibco), advanced DMEM/F12 (Gibco) were used. The methoxy PEG5000‐ PLGA (MW: 5000, mPEG‐PLGA) polymers were purchased from Xi'an Ruixi Biological Technology Co., Ltd., China.

##### Cell Viability and Cell Apoptosis Assay

For cell viability assay, cells were planted in 96‐well plate at the density of 6000 cells per well, and then cells were cultured for 24 h. After treatment with indicated drugs for 72 h, cell viability was detected by Alamar Blue Cell Viability Reagent (thermofisher) or Kinase‐Lumi ATP release assay (beyotime) as the manufacturer's instructions. For cell apoptosis assay, cells were planted in 6‐well plate at the density of 3 × 10^5^ cells per well. After treatment with indicated drugs for 72 h, cell apoptosis was detected by PI and Annexin V staining (keygentec, KGA105) as the manufacturer's instructions.

##### Immunostaining and Western Blot

For immunostaining, cells were treated with cisplatin, then fixed with 4% formaldehyde, and permeabilized with 0.5% Triton X‐100. After incubation with TOM20 (Santa Cruz Biotechnology), cell morphology was observed under the Zeiss LSM‐700 confocal microscope as previously described.^[^ ^11^ ^]^

For western blot, cells were harvested and lysed with radioimmunoprecipitation assay buffer containing protease inhibitors. Equivalent protein was resolved on sodium dodecyl sulfate‐polyacrylamide gel electrophoresis (10% gels) and transferred to nitrocellulose membranes (Bio‐Rad). Protein bands signaling was visualized as previously described.^[^ ^11^ ^]^

##### Assay to Detect Cellular Respiration

To detect mitochondrial oxidation capacity, real‐time oxygen consumption rate (OCR) was measured on a Seahorse XF24 Analyzer (Agilent Technologies, USA). Cells were plated in 150 µL complete DMEM growth media at 1  ×  10^5^ cells per well density onto 24‐well Seahorse plates (Agilent Technologies, USA) 24 h prior to the assays. The medium was removed and was replaced by glutamine‐ and pyruvate‐free DMEM medium (D5030 pH 7.4). The basal OCR was calculated by XF24 Analyzer software (Agilent Technologies, USA) after 1.5 h incubation in the DMEM medium. To detect effects of drug treatments on OCR, cells were treated with indicated drugs for 24 h, and basal OCR was calculated by XF24 Analyzer software.

##### Proteasome Activity

To detect proteasome activity, the same number of MDA‐MB‐231 and 231‐R3 cells were harvested and measured with a Proteasome Activity Assay Kit (Abcam) according to the manufacturer's protocol. In this case, cell lysis was incubated with Suc‐Leu‐Leu‐Val‐Tyr‐7‐amino‐4‐methylcoumarin (Succ‐LLVY‐AMC), and the free AMC fluorescence was measured on a fluorometric microplate reader.

Fluorescence was measured with a Multi‐Mode Detection platform (Molecular Devices) in the presence/absence of MG132 at 37 °C for 60 min. The increase of fluorescence (ΔRFU) at 350/440 nm was detected as proteasomal activity. ΔRFU = (RFU2 − iRFU2) − (RFU1 − iRFU1), RFU1 is total proteolytic activity at time 1, iRFU1 is nonproteasome proteolytic activity at time 1.

##### Protein Carbonylation

Cells were lysed, proteins were extracted and processed as Abcam's instructions using a Protein Carbonyl Content Assay kit (ab126287 and ab178020).

##### mRNA Isolation and Quantitative RT‐PCR

Cells were harvested and homogenized in Trizol solution (Invitrogen). RNA was extracted using the guanidinium salt/phenol–chloroform method, and the QuantiTect Reverse Transcription Kit (Qiagen) was used to prepare cDNA according to the manufacturer's protocol. Quantitative real‐time PCR was conducted using Taqman Gene Expression Master Mix (Life Technologies). The primers used in this study is listed in Table S5 in the Supporting Information.

##### Fabrication of Drugs‐Loaded mPEG‐PLGA NPs

Bortezomib‐loaded mPEG‐PLGA nanoparticles (Bort_na NPs) and cisplatin‐loaded mPEG‐PLGA (Cis_na NPs) were prepared via an optimized nanoprecipitation method based on tetrahydrofuran (THF)/water solution. First, 25 mg mPEG5000‐PLGA (Xi'an Ruixi Biological Technology Co., Ltd., China), 5 mg bortezomib and cisplatin powders were completely dissolved in 1 mL THF, respectively. Then, the mPEG‐PLGA and drug at a feeding ratio of 10, w/w% (to polymer) were mixed adequately in THF (200 µL). Then the solution was quickly added dropwise to 4 mL aqueous solution under sonication for 1 min. The ration of oil to deionized (DI) water is 20. The resulting mixture was stirred overnight open to air to evaporate THF and encapsulate the drugs. Next, the NPs were purified by filter centrifugation using Amicon Ultra‐4 filter (MWCO: 30 kDa) at 18 000 rpm for 15 min (Millipore, Billerica, MA, USA). The obtain products were washed three times with DI water to remove nonencapsulated drugs. Finally, the drug‐loaded mPEG‐PLGA NPs were lyophilized for further use.

##### Xenograft Experiments

MDA‐MB‐231 or 231‐R2 cells were injected into the right shoulder of female nude mice (1.0 × 10^6^ cells for each injection). When the xenografts became visible, the mice were randomly divided into indicated groups (five mice for each group). For the combination of bortezomib and cisplatin treatment, bortezomib and Bort_na were administrated every 4 days by intraperitoneal injection at 1 mg kg^−1^. Cisplatin and Cis_na were administrated every 4 days by intraperitoneal injection at 5 mg kg^−1^ 4 h after bortezomib or Bort_na treatment. Tumor volume and mice body weight were measured accordingly. Tumor volume was calculated as *V* = (*a* ^2^ × *b*)/2, where *a* is the width and *b* is the length of the tumor.

##### Ethical Approval and Consent to Participate

The design of the in vivo study was approved by the Animal Care and Use Committee of University of Macau.

##### Statistical Analysis

*RNA‐seq Analysis*: The quality of pair‐end reads was checked with FastQC (V.0.11.5). Then TrimGalore (v.0.4.5) was used to filter the low quality reads and remove adaptors. Next, clean reads were mapped to GRCh38 using STAR (v.2.5.3a) with default parameters. For the amount of read for each gene, HTSEQ (v.0.6.1) was applied to all samples. Reads only aligned to a unique position were considered, while excluded that mapped ambiguously. To compare the expression among genes in different samples, the expression of each gene was quantified as Fragments Per Kilobase of transcript per Million mapped read (FPKM).

*Image Analysis*: EBImage package (v.4.26.0) was used to count the number of cells in CFP and YFP images. The mean intensity of each cell in the image was calculated. And apoptosis cells were identified by YFP and CFP intensity ratio.

*GEO Dataset*: To estimate the graded reduction of mitochondrial respiratory activity among breast cancer patients, the expression data in breast cancer were downloaded from a cohort (GSE6434) including 24 breast cancer patients for further analysis. These patients were treated with docetaxel and had sensitive or resistant response. The normalized expression data of genes related to mitochondrial respiratory were extracted to construct heatmaps. And the correlation of mitochondrial respiratory genes expression between samples was calculated using Pearson method.

All cell culture assays were replicated three times. Graphs were generated by GraphPad Prism 8, and data were analyzed by Student's *t*‐test between two groups or two‐way analysis of variance (ANOVA) with Bonferroni's post‐test among multiple groups. All values were presented as mean value (three replications) ± SD. *p* Values were indicated by asterisks as followed: *p < 0.05 and **p < 0.01.

## Conflict of Interest

The authors declare no conflict of interest.

## Author Contributions

C.X.D. designed and provided guidance for this study. F.Y.S., X.Y.L., and J.L. acquired the data. C.X.D. and F.Y.S. wrote the manuscript. F.Y.S. and H.X. conducted the experiments. Y.L.D. and L.S.X. prepared for the nanoparticles, K.M., H.T.W., and Q.C. developed the methodologies. R.B.D. and P.C. provided the tumor organoids. X.Z. analyzed the data. S.E.M. conducted the RNAi screening. G.C. and N.W.L. provided the patient samples. G.K.C. contributed to the development of the 69‐drug library. G.Y.W. and F.Q.X. provided the caspase 3 (C3) biosensor reporter cells.

## Supporting information

Supporting Information

Supplemental Table1

Supplemental Table 2

Supplemental Table 3

Supplemental Table 4

Supplemental Table 5

## Data Availability

The datasets used and/or analyzed during the current study are available from the corresponding author on reasonable request. All data generated or analyzed during this study are included in this published article [and its Supporting Information files].
