# Supplementary material for: Enhanced Protein Damage Clearance Induces Broad Drug Resistance in Multitype of Cancers Revealed by an Evolution Drug‐Resistant Model and Genome‐Wide siRNA Screening
Source: Adv Sci (Weinh). 2020 Oct 11;7(23):2001914. doi: 10.1002/advs.202001914 (PMC7709997; doi:10.1002/advs.202001914)
Supplement: Supplementary file 1 — Supporting Information [file ADVS-7-2001914-s001.pdf]

© 2020 Wiley-VCH GmbH

ADVANCED SCIENCE
Open Access

Supporting Information

for *Adv. Sci.,* DOI: 10.1002/advs202001914

Enhanced protein damage clearance induces broad drug resistance in multi-type of cancers revealed by an evolution drug resistant model and genome-wide siRNA screening

*Fangyuan Shao, Xueying Lyu, Kai Miao, Lisi Xie, Haitao Wang, Hao Xiao, Jie Li, Qiang Chen, Renbo Ding, Ping Chen, Fuqiang Xing, Xu Zhang, Guang-Hui Luo, Wenli Zhu, Gregory Cheng, Ng Wai Lon, Scott E. Martin, Guanyu Wang, Guokai Chen, Yunlu Dai and Chu-Xia Deng**

The description of the supporting information is included in the manuscript.

Supplementary Figures

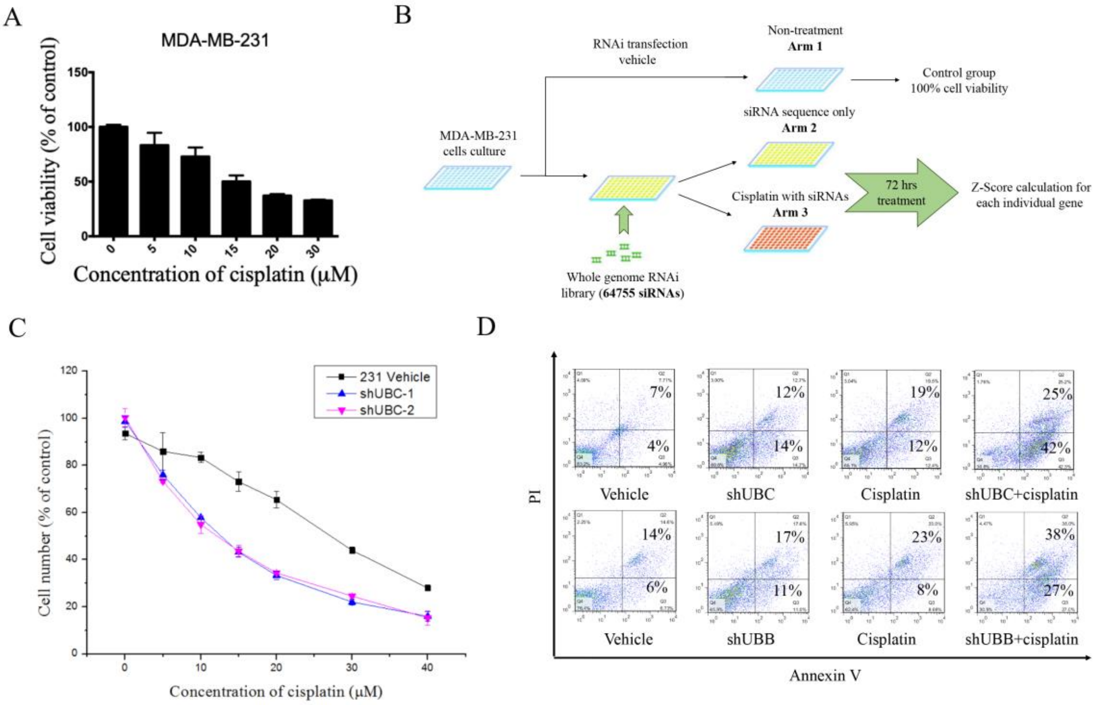

Supplementary Figure 1. Genome-wide RNAi screening identifies proteasome as a target for enhancing cisplatin efficacy. (A) MDA-MB-231 cells were treated with different concentration of cisplatin, and cell viability was detected by alamar blue assay and 10 μM cisplatin achieved about 30% killing effect, which is selected for RNAi screening. (B) Flow chart of the RNAi screening assay. (C) MDA-MB-231 cells were transfected with UBC shRNAs before cisplatin treatment for 48h, and cell viability was detected by alamar blue assay. (D) MDA-MB-231 cells were transfected with UBC or UBB shRNAs before cisplatin treatment for 48h, and cell apoptosis is detected by Annexin V and PI staining.

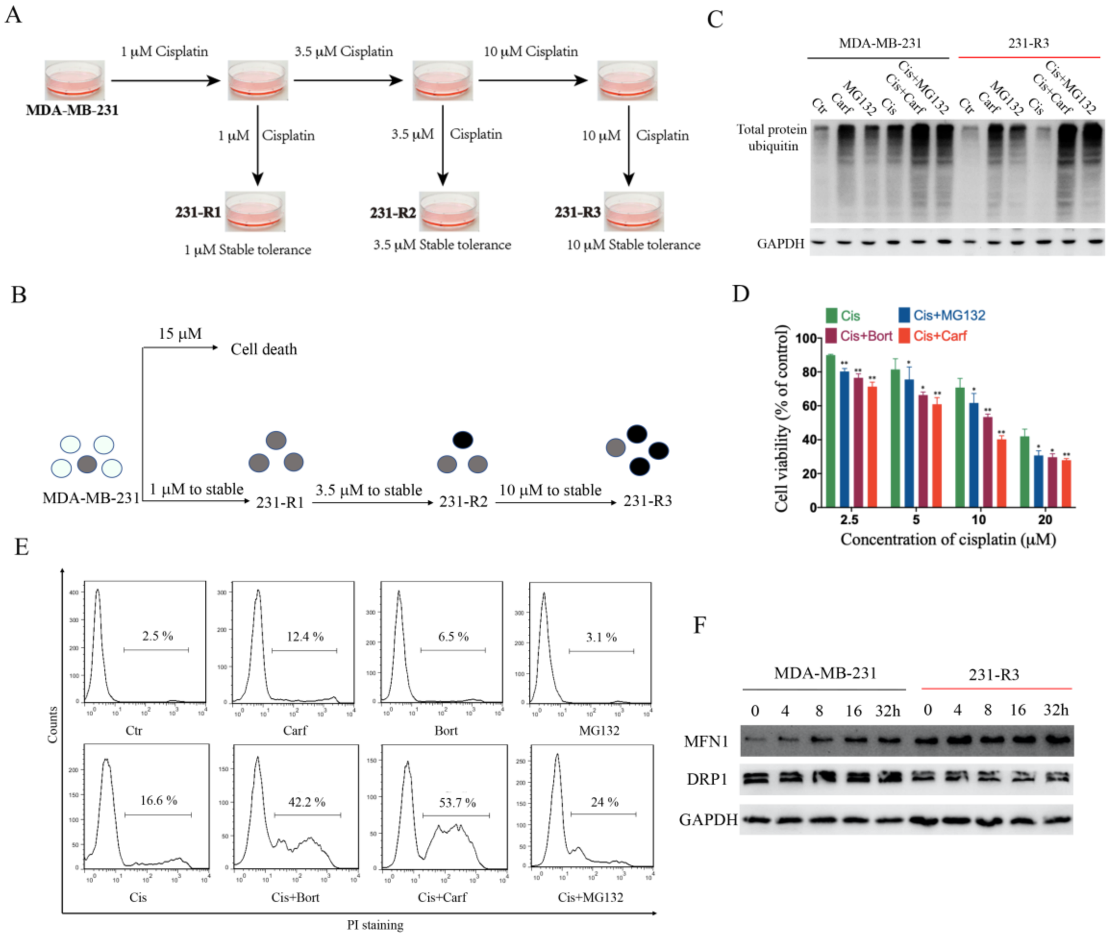

Supplementary Figure 2. Establish acquired resistance through an evolution process by gradually increasing concentrations of cisplatin. (A) Flow chart of developing cisplatin resistant cell lines (231-R1, 231-R2, and 231-R3) from MDA-MB-231 cells. (B) Urgent treatment of 15 μM resulted in cell death, and initial low dose of cisplatin selectively enriched cells with tolerable changes. Then graded increase of drug stress and eventually enable cells became fully resistant. (C) MDA-MB-231 and 231-R3 cells were treated with cisplatin alone or combined with proteasome inhibitors, and protein total ubiquitin was detected by western blot. (D) MDA-MB-231 cells were treated with cisplatin alone or combined treatment with proteasome inhibitors, and cell viability is detected by ATP production assay. (E) MDA-MB-231 cells were treated with cisplatin alone or combined treatment with proteasome inhibitors, and cell death is detected by PI staining. (F) MDA-MB-231 and 231-R3 cells were treated with cisplatin as indicated time, and expression of MFN1 and DRP1 were detected by western blot.

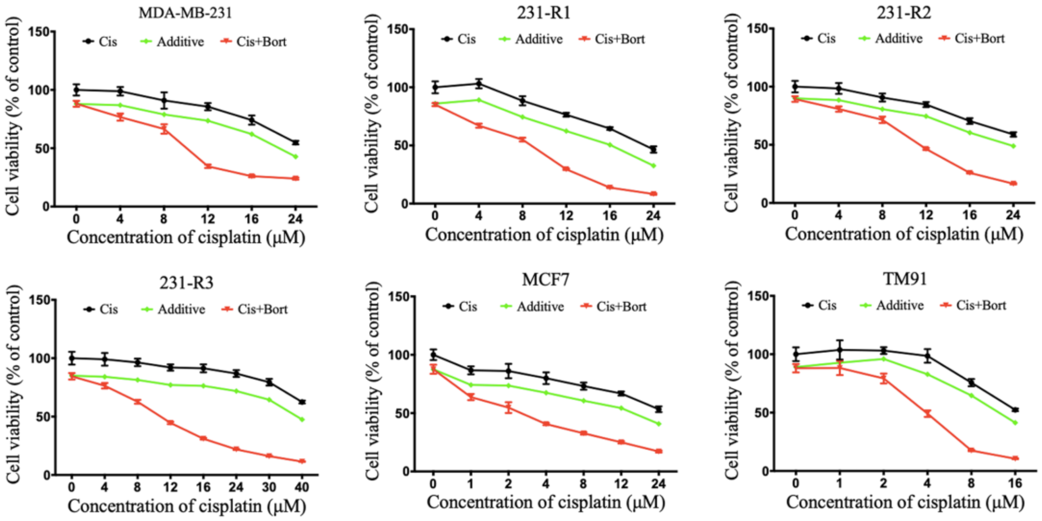

Supplementary Figure 3. Combined treatment with bortezomib treatment to enhance cisplatin efficacy in multiple breast cancer cell lines.

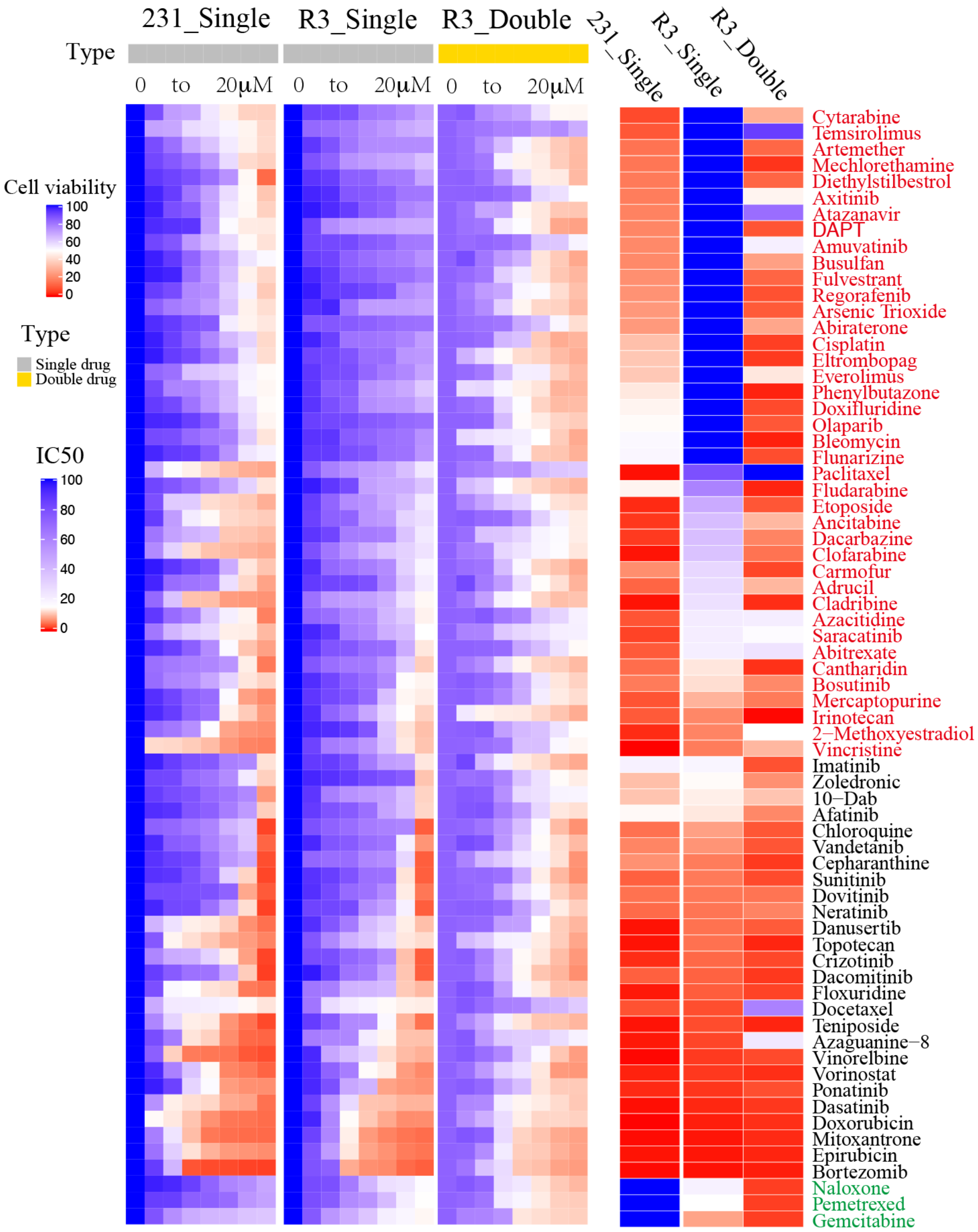

Supplementary Figure 4. 69 Drug screening in MDA-MB-231 and 231-R3 cells and combined with bortezomib treatment in 231-R3 cells, showing cell viability and drug IC50, respectively. MDA-MB-231 and 231-R3 cells were treated with 69 drug library, and 231-R3 cells were found to be resistant to 40 drugs (Red), 26 drugs have no significant difference in both cell lines (Black), and 3 drugs were more effective in 231-R3 cells (Green).

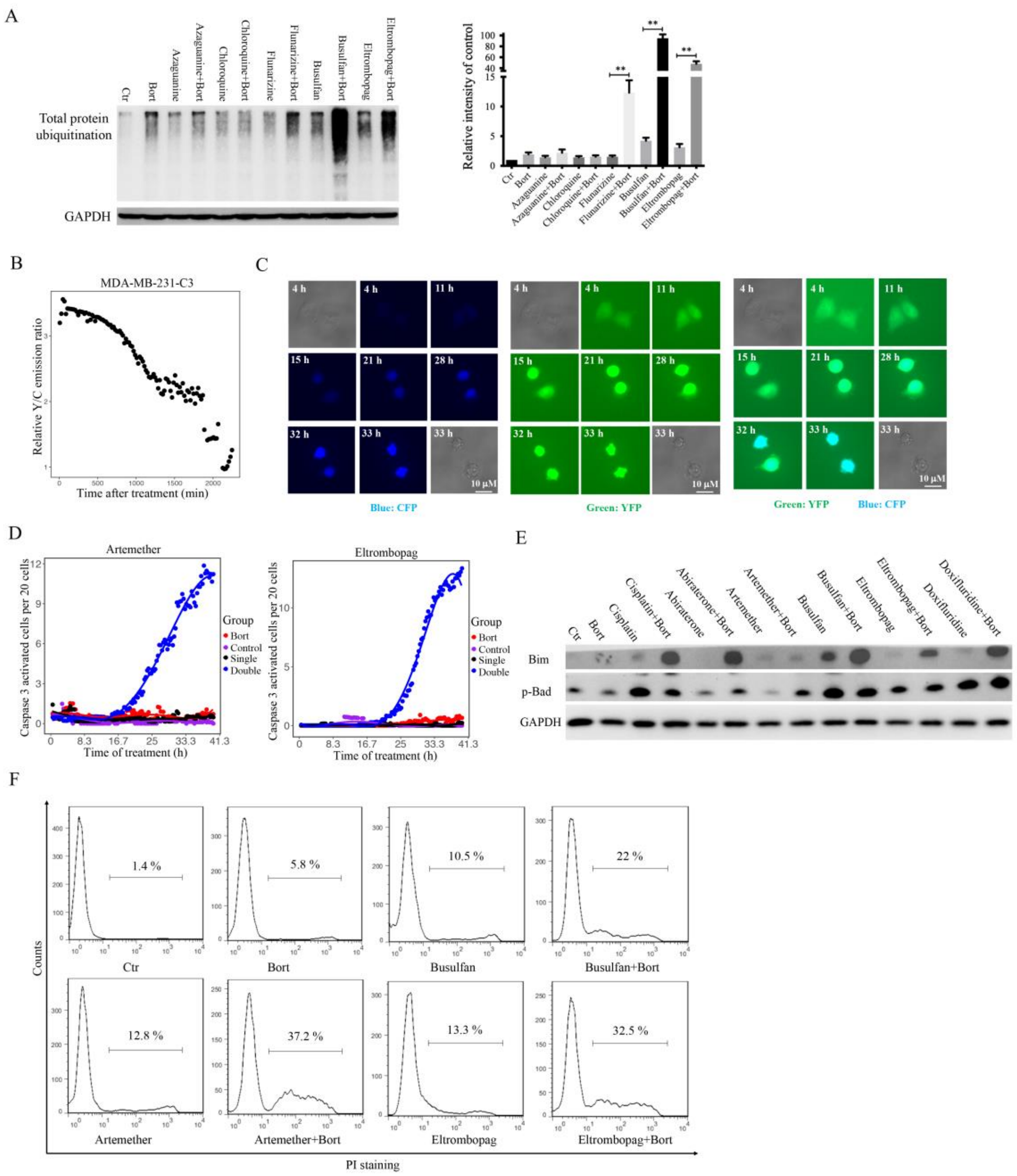

Supplementary Figure 5. 69 Drug screening with bortezomib in MDA-MB-231 and 231-R3 cells and FRET- based caspase- 3 (C3) Biosensor system. (A) MDA-MB-231 cells were treated with indicated drugs, and protein total ubiquitin was detected by western blot and gray value were measured for each group of three independent assay. (B) FRET- based caspase- 3 (C3) Biosensor labeled MDA-MB-231 cells are treated with cisplatin, and dynamic decrease of the YFP to CFP ratio were monitored in 4 individual apoptosis cells. (C) Dynamic changes of the phase image and the fluorescence images of YFP and CFP from two neighboring apoptosis cells, which were treated with cisplatin. (D) FRET- based caspase- 3 (C3)

Biosensor labeled MDA-MB-231 cells were treated with indicated drugs or in combination with bortezomib treatment, and dynamic change of caspase 3 activation were monitored during 50h treatment for each group. (E) 231-R3 cells were treated with indicated drugs and combined with bortezomib treatment, and Bim and phosphorylated Bad were detected by western blot. (F) MDA-MB-231 cells were treated with indicated drugs or combined with bortezomib treatment, and cell death is detected by PI staining. All values are presented as mean value (three replications) ± SD, *P < 0.05 and **P < 0.01. (A, two-tailed Student's *t*-test.)

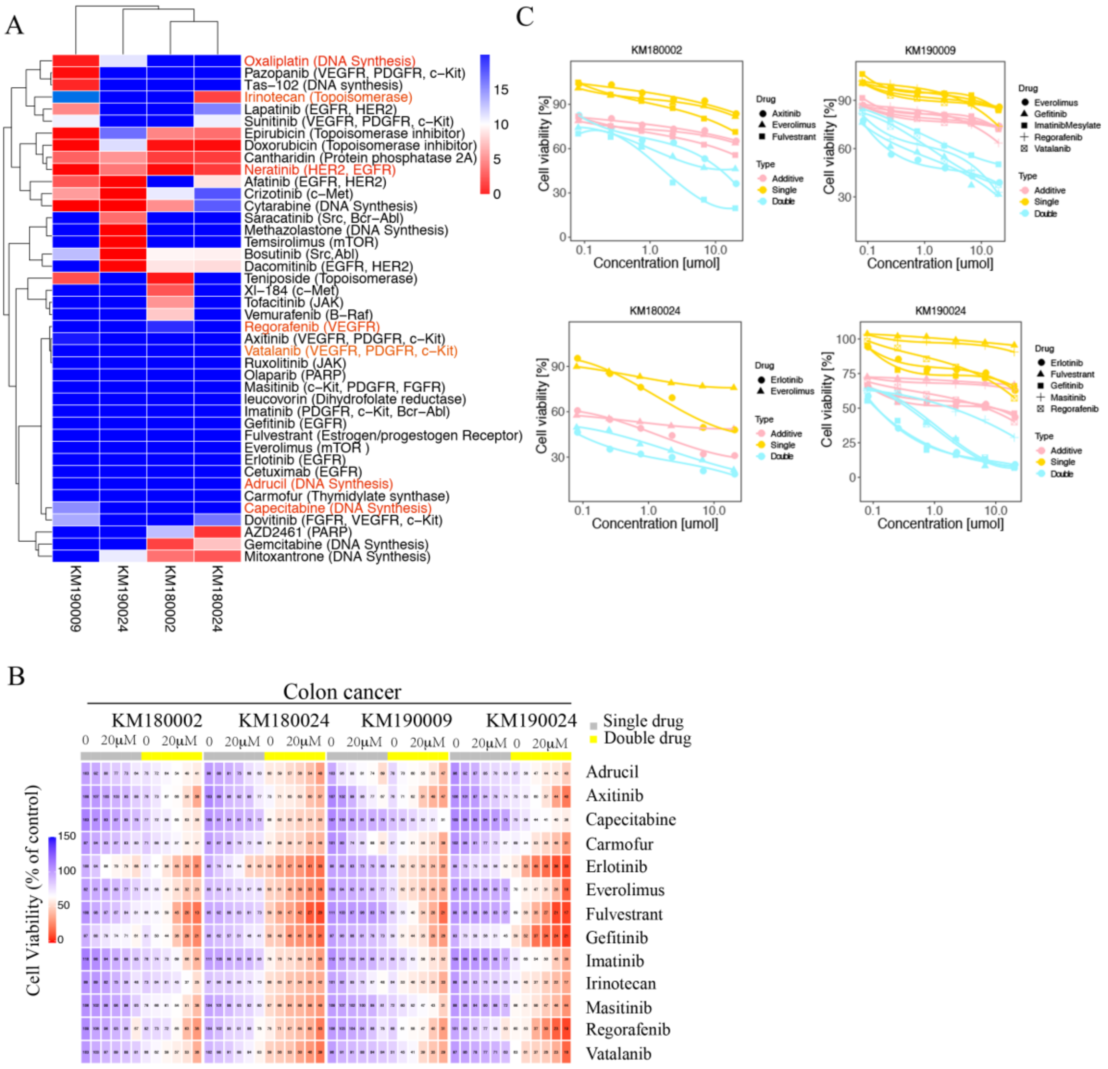

Supplementary Figure 6. Combined treatment of different anticancer drugs with bortezomib for individual colon cancer patients. (A) IC50s for 42 drugs screening in patient derived organoids for 4 colon cancer patients. (B) 13 resistant drugs were tested

in combination with bortezomib treatment in 4 colon cancer patients, and cell viability were shown for single drug treatment and double treatment, respectively. (C) Drug synergy analysis for 4 colon cancer patient samples.

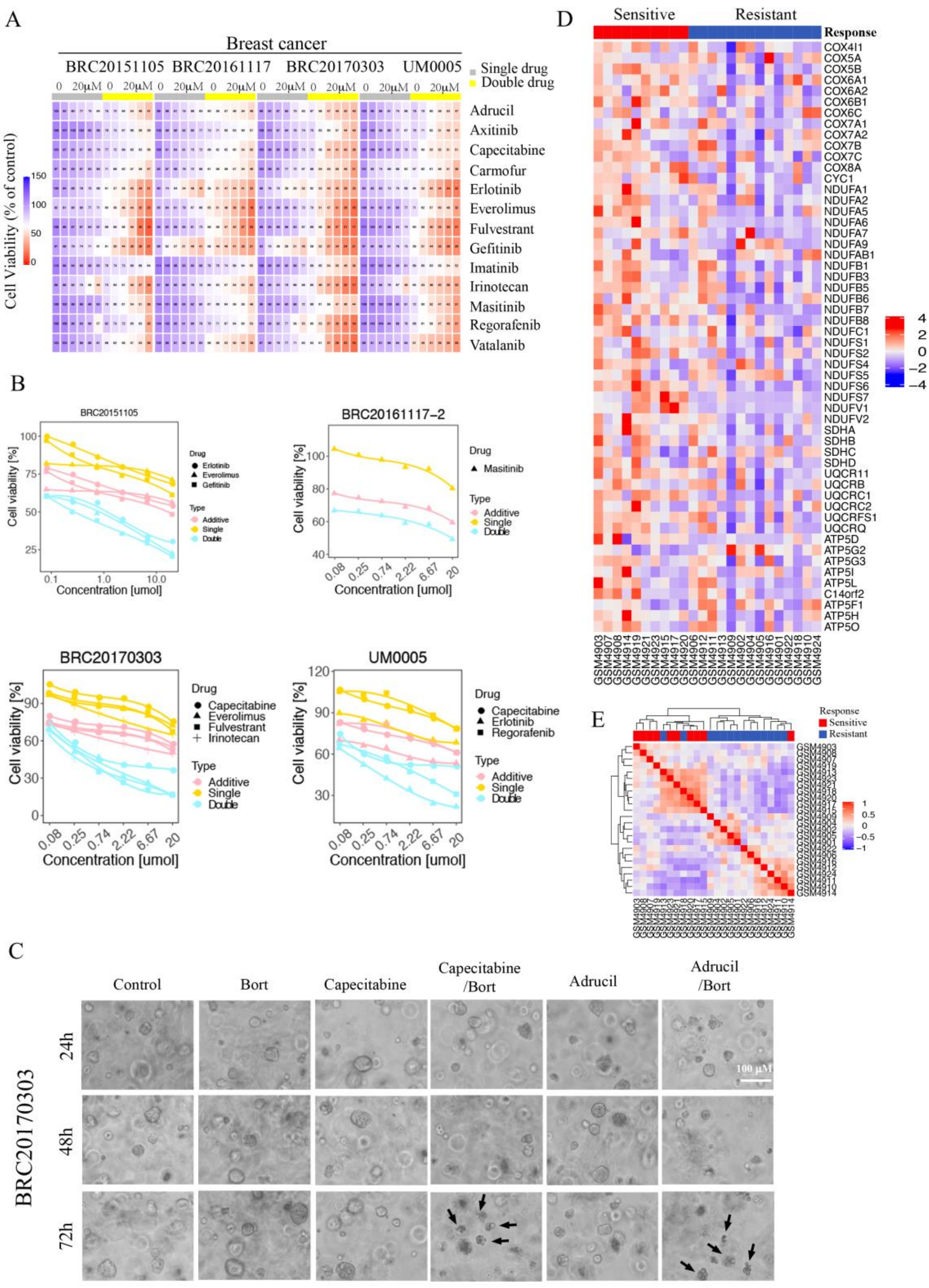

Supplementary Figure 7. Combined treatment of different anticancer drugs with bortezomib for individual breast cancer patients. (A) 13 resistant drugs were tested in combination with bortezomib treatment in 4 breast cancer patients, and cell viability

were shown for single drug treatment and double treatment, respectively. (B) Drug synergy analysis for 4 breast cancer patient samples. (C) Breast cancer patient' samples BRC20170303 was treated with indicated drugs, and typical dead spheres were shown in triple treated group. (D) Heatmaps of gene expression related to mitochondrial respiratory complex (n=54) in breast cancer patients (n=24) between resistant and sensitive group. (E) Correlation heatmaps for breast cancer patients (n=24) of mitochondrial respiratory activity genes (n=54).

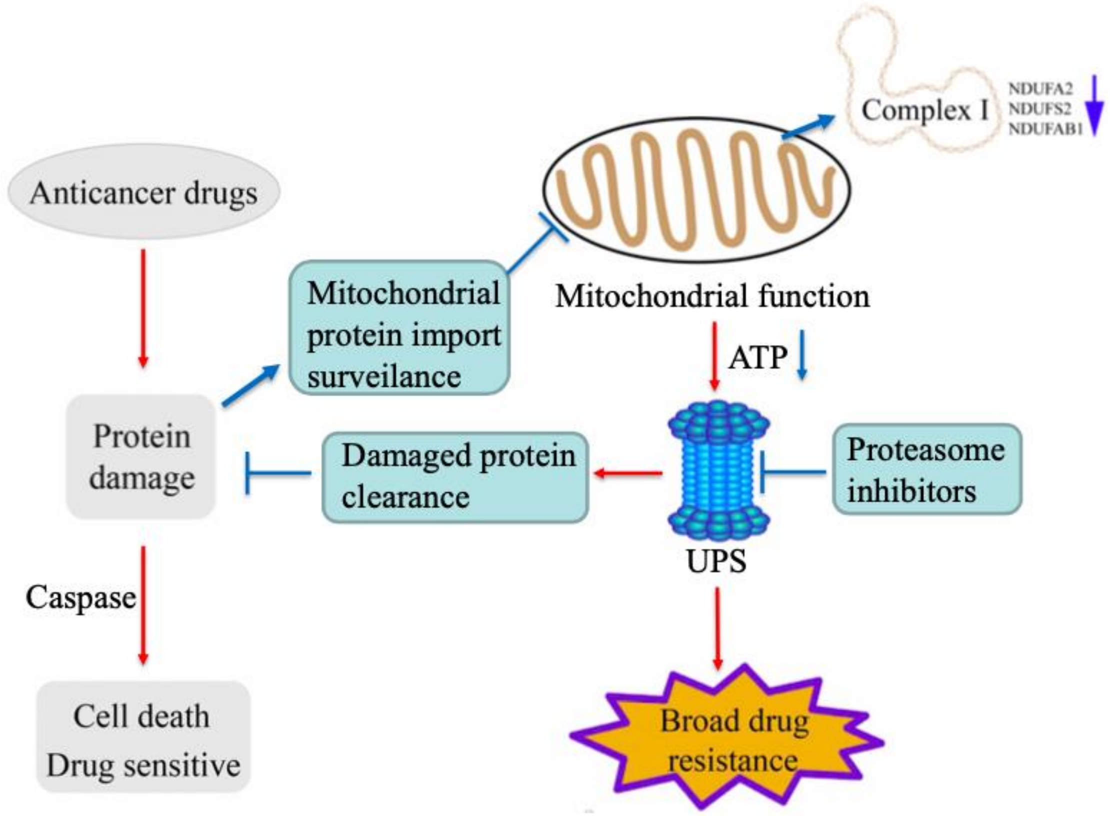

Supplementary Figure 8. Genome wide siRNA screening and evolutionary drug stress studies identified mitochondrial respiration and proteasome activity as key component for broad drug resistance, which could be eliminated by bortezomib treatment. The blue arrows contribute to broad drug resistance, and the red arrows contributed to drug sensitivity.
